# Experiments with Pental

**Published:** 1892-06

**Authors:** 


ARTICLE VII.

### EXPERIMENTS WITH PENTAL.

**Extracts from communication of Dr. Ludwig Hattgassy, Assist-at Dental Clinic of the Royal University of Buda-Pesth.**

In my experiments, the following questions were the subjects of my special attention :

What is the narcotic influence of pental ?

When does the narcotic state commence, and how long does it continue ?

How does the nervous system bear this remedy ?

What is its influence on the heart and the respiratory organs ?

What are the after-effects of the remedy ?

There is no doubt that pental is an energetic and quickly acting anæsthetic. From the inhalation till the narcose, when the operation can commence, it takes on the average 1¼ minutes. There were cases when the narcose was so profound after ½–¾ minutes, that the operation could be then begun.

The quantity of pental necessary to produce narcose cannot be precisely estimated, as it seems to depend on the individual disposition. The smallest dose which produced narcose, was three grains, the average quantity 6-8 grains, and the largest doses 12-15 grains; a larger one than the latter was seldom used.

To determine the narcose, three circumstances are to be observed. First, the condition of the pupil. In seventy per cent. of the cases the eyes are open, in the others the eyes remained open even after lifting the lids, so that we can continually observe the pupil; by this we learn that during the narcose the pupils are always more dilated, and the narcose occurs at the middle dilation. The pupils either do not react at all against light, or only minimally. When, therefore, the light reaction does not take place, when pupils are dilated, the narcose is sufficiently deep for the operation.

A second symptom is the insensibility of the skin. When, for instance, we pinch the underarm, and no reaction takes place, we are near the time to commence the operation, but it is always better to continue the inhalations five or six times after this test, to make it certain.

My experiences about the blood-vessels were, that in twenty per cent. of the cases observed by me the pulse remained uniform quantitatively as well as qualitatively, wherefore I have also accepted the action on the heart in these cases as unimportant. In some of the other cases no great changes took place, yet the beating of the pulse was both rarer and weaker, in others, the pulse was slower soon after the inhalation, which often was followed by a quantitative increase. Very seldom I have seen a light cyanose, and never so prominent as it occurs with laughing gas. It was evident, however, from all observations, that this agent has, in some individual cases, a strong effect on the heart, so that this must be carefully watched.

Concerning the duration of the narcose, if it be a superficial one, the patients wake immediately after the operation; if the narcose be a deep one, they remain in a soporous condition for one to two minutes. Sometimes the awakening takes place after five minutes, and then the patient remains soporous for a long time.

Of the after-effects we are quite satisfied. Even where organic disease exists and pental was adminstered, the patients bore well the narcose.

During the operations, it is absolutely necessary that the chest be free from all clothing whatever; and it is also necessary that an assistant be present during the narcotic state, not only for the sake of making observations, but also because the excitement stage of the unconscious individual is often so violent, that one person can not subdue it.

In conclusion, it must be admitted that pental is a strong remedy, which acts with intensity on the heart as well as the nervous system, and it cannot be recommended in private practice in larger operations. The indications are, however, that in operations requiring the least time, and with skillful manipulation, pental is preferable to either laughing gas or bromether.—*Items of Interest.*

---

